# Reasoning COVID-19: the use of spatial metaphor in times of a crisis

**DOI:** 10.1057/s41599-022-01264-8

**Published:** 2022-08-08

**Authors:** Dominik Kremer, Tilo Felgenhauer

**Affiliations:** 1 Friedrich-Alexander Universität Erlangen-Nürnberg, Erlangen, Germany; 2 Pädagogische Hochschule Oberösterreich, Linz, Austria

**Keywords:** Geography, Language and linguistics

## Abstract

As other crises before, the COVID-19 pandemic put established discursive routines at stake. By framing the pandemic as a crisis, an immediate search for adequate counter-measures started to define proper means of mitigation and protection for the population. In the early stages of COVID-19, when little reliable information on the virus and its transmission behaviour was available, an intense use of metaphor to explain and govern the crisis had to be expected. Beside its well-known impact on (geo-)politics, a thorough analysis especially of the use of spatial metaphors to reason about the crises is still missing. In our approach, we rely on the foundational work of Lakoff and Johnson (1980) on image schemata, and prior work on spatial metaphors as part of argumentation patterns from cultural geography (Schlottmann, 2008). After a thorough analysis of prominent examples according to the argumentation scheme of Toulmin (1976 [1958]), we explored examples from the pre-existing corpus on COVID-19, deliberately compiled by DWDS for analysis of language patterns used throughout the pandemic. In a subsequent filter-refinement approach building on methods from cognitive linguistics and utilising a chunk of the same corpus, we were able to obtain and discuss results on the variety of spatial metaphors used at that time.

## Background

### Geography’s linguistic turn. From spatial essentialism to linguistic reconstruction

Since the 1970s, a turn to the humanities, including e.g. hermeneutics, phenomenology, social and cultural theory, took hold of geography, puzzled and stimulated theoretical discussion and enriched its range of perspectives and methodological approaches. The “linguistic turn” was received within geography in the context of constructivist theory: space and place were no longer considered to be simple physical entities but, instead, they were the outcome of complex societal processes, actions and structures; in short: space and place were considered to be cultural, social, and, at its core, linguistic constructions. For example, Barnes and Duncan (1992), inspired by literary studies, claimed in their influential volume “Writing Worlds”: “For what is true is made inside texts, not outside of them…” (Barnes and Duncan, 1992, p. 3) – a clear critique of traditional geography and its unreflected spatial essentialism. Duncans and Gregory’s “writes of passage” (1999) followed a similar argument: in the reconstruction of historic travel writing the meaning of space and place, not only its obvious physical and material appearance becomes visible. In his renewal of cultural geography, Peter Jackson shifts his focus towards “maps of meaning” as he perceived - among other subjects - the critique of “languages of racism” to be constitutive for a “new cultural geography” (Jackson, 1989, pp. 132ff). Werlen (2007, pp. 343ff), exploring the potential of Anthony Giddens‘ theory of structuration for social geography, coined the phrase of “symbolic regionalisation” to refer to the manifold ways of linguistic and visual constructions of space as did Anssii Paasi’s research on regional identity (Paasi, 1991). Given this background, the path towards a more detailed analysis of the relationship between language and space was marked; several strands of linguistics became a source of methodological innovation in human geography, especially for qualitative empirical research in human geography.

In the last decades linguistic constructivism has been both contested and informed by “non-” and “more-than-representational theory”, a “new materialism” (Lorimer, 2005; Whatmore, 2006; Thrift, 2007) and “post-phenomenology” (Ash and Simpson, 2016) – which appears at first sight as a simple counter-balance in order to reinstate some kind of “material”/“ideal” equilibrium. But more interestingly, it has extended the view of geography’s linguistic constructivism towards concepts of practice, event, performance, presence, and experience. These concepts have in common to shift the geographic understanding of language from static word-object-reference (in fact, the linguistic representation of an assumed “pre-representational” world) towards a generic understanding of language and space. Daya (2019, p. 373) observes “…a shift away from the notion of representation as signification to an understanding of texts and images as generative…” – a perspective which is already present in the philosophy of language and in action theory, namely as the foundation of language in bodily experience (image schema), the performance of discoursive power (e.g. the naming of things as the creation of things) and, most explicitly, in the pragmatist concept of the “speech act” (Austin, 1962).

**Fig. 1:**
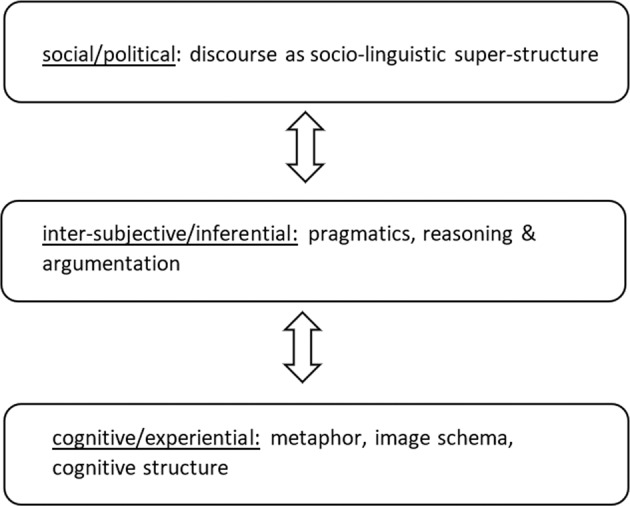
Argumentation and reasoning acting as hinge between cognitive linguistics and discourse analysis. Discourse as socio-linguistic vantage point is not fully compatible with methodology from cognitive linguistics. In adding the layer of pragmatics, reasoning & argumentation that act as hinge, we conceptually overcome this shortage. This figure is covered by the Creative Commons Attribution 4.0 International License. Copyright © the authors of this paper, all rights reserved.

In the following, the geographical reception of three linguistic paradigms will be described in more detail: cognitive linguistics, discourse theory and analysis, and language pragmatics (see Fig. 1 and Table 1).

**Table 1:** Linguistic perspectives, spatial aspects and COVID-19-references.

Linguistic analytic dimension	Correspondent social/spatial aspects	Geographical examples	Examples from the COVID-19 discourse
Discourse theory/analysis	Power structures in the genesis of hegemonic discoursive meanings/power-related framing of societal issues	Framing/imagining cultural differences as spatial differences; recurring patterns of geopolitical world views (e.g. “rogue states”; “clash of civilisations”)	Labelling COVID as “China-virus”; establishing frameworks for mapping and surveying the global spread of COVID
Pragmatics, reasoning, argumentation	(Spatial) reasoning as social action; interplay of argumentational logics and social rules; interplay of explicit statements and implicit assumptions and inferences	Justification of statements in reference to spatial entities; e.g. acting in response to/in the name of territories	Argumentative use of metaphors and established discourse patterns in political rhetorics: e.g. suggesting the “inevitable” need for spatially defined social restrictions
Cognitive linguistics	Metaphors in relation to the human body and its spatiality; e.g. inside-outside-/up-down distinction as basis for human cognition	Container-metaphors implicit to the social construction of national territory	Containment & containerisation in descriptions/definitions of the pandemic

According to the theoretical framework introduced (see Fig. 1) we provide an overview of correspondent social/spatial aspects, geographical examples and examples from COVID-19 discourse.

### Language and space in cognitive linguistics: metaphors and image schemas

Cognitive linguistics proved to be a vital source for social geography’s interest in language and its spatial implications. Lakoff and Johnson’s (1980) groundbreaking work on metaphors and the redefinition of their role for human life revealed both the importance of spatial thinking for everyday language practice as well as the relevance of cognitive linguistics for geographical thinking. Conceptual metaphors, or put more fundamentally, so-called “image schemas”^1^, are not secondary aesthetic ornaments of language or sophistic means taken from the rhetorical “toolbox”. Instead, they form a basic set of frameworks for our cognition, imagination, and, thus, our language practice:

“Image schemas are relatively simple structures that constantly recur in our everyday bodily experience: CONTAINERS, PATHS, LINKS, FORCES, BALANCE, and in various orientations and relations: UP-DOWN, FRONT-BACK, PART-WHOLE, CENTER-PHERIPHERY, etc.” (Lakoff, 1987, p. 267).

Knowledge and common beliefs on the existence and shape of place and space are rooted in bodily metaphors in our thinking and speaking. Geography’s usual subjects such as place, territory, region, or landscape can be derived from their origin in human cognition and in linguistic metaphors or image schemata. Schlottmann (2008) highlighted the function of the container-metaphor in making sense of the spatial world:

“…the container concept seems to be one of the most common ways of referring to a surrounding world. Spatial entities, for instance, are normally imagined and treated as vessels that contain various objects (people, matters, resources, etc), or as boxes in which objects are (or shall be) placed” (Schlottmann, 2008, p. 828).

Schlottmann showed in her analysis of the language use on Germany’s unification that despite the frequently repeated rhetorics of unification the general assumption of separated spatial containers (West-Germany/East-Germany) remained intact. The cognitive structure of spatial imagination constantly reproduced language patterns of separation (ibid., p. 831). In addition, Weichhart and Weixlbaumer (1988) and Shortridge (1985) were able to show that place images prove to be very stable and independent from material changes in the “real world”. Thus, sociocultural concepts are more likely to change their spatial reference than their associated semantics, if the living environment of the specific place changes. Further, the centre-periphery schema is a recurring implicit element of the medial construction of regional identities. In putting the adressed region in the imaginative centre of space a sense of originality, prominence and superiority is assigned to the promoted region (Felgenhauer et al., 2005, p. 55). Various techniques of “othering” can easily rely on such patterns in the sense that they align spatial with social distinction.

Potential conclusions from empirical work on spatialisation and containerisation were intensively discussed within human geography (Schlottmann, 2008, pp. 823ff). On the one hand, increasingly globalised and networked spatial “relational” realities have to be reflected in a modified everyday language use and, thus, the language of static containers would have to be overcome - both in science and society. On the other hand, the function of containerisation for reducing complexity and making sense of the world has to be acknowledged and cannot be changed easily.

### Discourse analysis on space and place

Discourse theory and analysis made a profound contribution to our current understanding on how language, society, and politics are intertwined (e.g. Kendall and Wickham, 1999; Chilton, 2004; Wodak and Chilton, 2005). The power of hegemonic discourse patterns, of strategic wordings and the explicit and implicit discoursive framing of societal phenomena and problems represent both, the possibilities and boundaries of social interaction. While theoretically contingent in the sense of linguistic constructivism, discourse patterns often prove to be highly stable and effective power structures – not only with regard to the often highlighted issues of class, race, and gender. Especially, spatial and territorial definitions, distinctions and oppositions remain a vital element in ever changing social and political environments on every scale level (e.g. Glasze, 2013; Mattissek, 2008; Rheindorf and Wodak, 2018). For example, local and regional identities, nationalist ideologies, geopolitics, and imaginations of global development depend in their very existence on both vital and stable discoursive structures. Thus, geography’s perception of discourse theory and methodology has intensified since the beginning of the 21st century, especially in Germany. Examples originate from various sub-disciplines, from urban, social, cultural and political geography. For example, Mattissek (2008) analysed the neoliberal city-marketing strategies of Frankfurt, Cologne and Leipzig and their attempts to promote a particular framing of the urban discourse. The global cultural politics of France and the construction of a “francophone world” were subjects of extended research conducted by Glasze (2013). Marxhausen (2010) investigated the discoursive formation of European identity, as it is promoted in education policies and in public narratives of the cultural and spatial “essence” of Europe.

Discourse theory and analysis were originally seen as a counterpart to the perspective of cognitive linguistics. However, in the current linguistic discussion “discourse” and “image schema” are no longer to be treated as mutually exclusive theoretical approaches. Instead, connections between cognitive approaches in general (Wodak, 2006) and cognitive metaphor theory (CMT, see Hardie et al., 2007) in particular have long been explored to contribute in the context of critical discourse analysis (CDA, see Semino et al., 2004), work continued by Musloff (2012) and Hampe (2017). Perspectives on the interplay between metaphor/image schema on the one hand and discourse on the other are also provided by language pragmatics, as it can be seen in the example of (spatial) argumentation and reasoning.

### Speech act, pragmatics, and argumentation

Everyday language use and its relevance for the construction of (social) reality has been fully acknowledged by Wittgenstein’s (1953) pioneering philosophy of language and the more specific theoretical outline of the matter by John Austin’s concept of “speech act” (Austin, 1962). The long-standing opposite of “doing” (primary status) and “speaking” (secondary status) was transcended by showing how words do not primarily represent the outside “non-” or “pre-linguistic” world in the sense of unidirectional reference. Instead, words, combined in the unit of “speech acts”, contribute to a complex web of meaning construction through implicit expression, inference and internal linguistic logics (e.g. Davidson, 2009 [1983]; Searle, 1969). Philosopher Robert Brandom (2000) has renewed this perspective. According to his concept of “inferentialism” language use is characterised by social commitment, entitlement, and constant mental “score-keeping” on the ontological status which people assign to things by everyday language use. In doing so, people constantly have to admit to implicit meanings in order to make explicit statements (as they wish them to be understood and accepted by others).

This field of everyday practice has also been addressed by contemporary argumentation theory. According to Toulmin (1976 [1958]), argumentation theory and analysis is not so much about assessing the objective logic quality of a particular argument. Instead, the use of the argument in a social context has to be interpreted in order to understand both its internal logics and its discoursive relevance and meaning. In his well-known schema (see examples below), Toulmin considered an argument to consist of a *Claim* (or Conclusion) which is supported by *Data*. The *Warrant* is the rule which explains why the Data is suited to support the Claim. The so-called *Backing* collects general conditions and principles on which the Warrant depends. Thus, as argumentation practices combine explicit statements and implicit assumptions they interlink cognition and discourse (see, Fig. 1).

Argumentation analysis has been applied by human geographers with regard to subjects as diverse as regional identity (Felgenhauer, 2010), the appropriation of urban environments (Kremer, 2013), or energy production (Faller, 2016). The schema can also be applied to statements taken from German media discourse on the pandemic (DWDS^2^) which are presented in the following.

Detail analysis 1^3^: If we know how the Virus is acting, we have to do everything to slow down the spread of the virus (Bavarian Minister Herrmann; Merkur, 2020). 

Detail analysis 2: To close off whole cities and regions is more effective, because, then, non-symptomatic people also reduce contacts to other people. (DWDS/Tagesspiegel, 2020). 

Furthermore, with regard to argumentative reasoning about spatially organised information, we have knowledge of several productive patterns from the fields of cognitive geography and computer science. For example, place descriptions are bound to anchor concepts in communications that are expected to be common sense (Couclelis et al., 1987). Second, spatial and spatialized knowledge is composed of fragments from own experiences, visualisations and communications, coined *mental collage* by Tversky (1993). In this mental collage, spatial concepts range from the level of deep grounding (e.g. on wayfinding task, see Richter et al., 2013) to aggregated and fluid levels of uncertain regions (e.g. on evaluating unsafety, see Redepenning et al., 2010; Moura de Sousa et al., 2022).

Although we recognise the impactful work that has been spent on the topic of metonymy, following Littlemore (2015, p. 14), we distinguish metaphor from metonymy by involving cross-domain-mapping in the sense of Lakoff (1987). In our work, we focus on cross-domain-mappings utilised for the purpose of deliberation in argumentative structures. Interestingly, in confirmation between findings from both cognitive (Delafield-Butt and Trevarthen, 2015) and social sciences (new materialism, Farnell, 1996), there is strong evidence that reasoning is not exclusively bound to mere logic of thought, but massively accelerated by bodily enactable mental models. Lakoff and Johnson (1980) illustrate that on the following example:

“France fell into a recession and Germany pulled it out.”

While such reductions of complexity are often framed as metonymy, as stated above we read such figuration as metaphors that sketch an easy to imagine bodily relational space. As France and Germany both act as persons, the abstract process of a recession is figurated as something one could fall into, e.g. a hole or trap. The crises itself is thus framed as an accident that requires external help. Transforming reasoning into bodily enaction thus creates the image of a passing by first responder helping out.

Additionally, human reasoners are known to routinely change scales by spatial specialisation and generalisation (Richter et al., 2013).

Source 1: “What is the bus from Bielefeld doing here? They should go home, in Lower Saxony there is corona!” (Kremer, 2020)

In this example, simplified reasoning by generalisation takes place on several levels: (1) A bus with a licence plate from Bielefeld is personified (to carry people from Bielefeld). (2) The German federal state of Niedersachsen is known to have reported a high number of COVID-19 infections lately. (3) Bielefeld is thought to be a city in Niedersachsen^4^ (generalisation by zooming out). From these constituents the consequence is derived that people from that bus would put a not acceptable risk to people at their current location (cf. Kremer, 2020).

To conclude this short overview on the geographic work on language and space, the three linguistic perspectives (see Fig. 1) can be applied to the example of spatial metaphors in the COVID-19 discourse.

*Discourse theory and analysis* focusses on social/political power structures which establish hegemonic interpretations of the pandemic. For example, as reflected in the term “China-Virus”, coined by the US administration, it becomes not only an inherently spatial phenomenon. The term also determines how the pandemic is mapped and represented on a global scale (critical: Zhang and Xu, 2020). *Spatial argumentation and inter-subjective/inferential reasoning* combines cognitive metaphors and well-established hegemonic discoursive meanings. As the two examples above have shown, political actors claim their decisions to be suitable or even “inevitable” by referring to spatial assumptions. With regard to *cognitive linguistics* and bodily experience the container metaphor provides a basic pattern for framing the pandemic in a semantic as well as in a practical sense (means of containment). Again, the overview emphasises the role of argumentation and reasoning as the “interface” of cognition and public discourse. In doing so, it places cognitive concepts within the context of evident language use and practice as well as it might extend the focus of discourse analysis from power and hegemony towards the inherent inferential structure of particular statements. The individual perspective of each of the three paradigms and the corresponding geographical aspects with regard to COVID-19 are outlined in Table 1.

In our explorative prestudy (see Section “Explorative Pre-study”) we focussed on spatial metaphors as the main component of spatial argumentation. They combine cognitive/experiential and argumentative/inferential practices. Within the German media discourse on corona patterns of containerisation, personification and naturalisation proved to be most relevant.

### Current research on metaphors and COVID-19

Metaphors of crises are a well-known topic, so that we can relate to earlier work to frame our expectations on our empirical results. As a side note, the term crisis, originally used for a deciding moment in the course of a disease, was used to indicate a ‘turning point’ in general (Hülk, 2014). Whereas the term ‘catastrophe’ indicates severe life-threatening consequences on society which cannot be mitigated by individual action, “apocalypse” refers to a global inevitable scenario of loss of human life. ‘Crisis’ is thought to be an event that can be governed in the existing structure of society with a certain effort (Lickhardt and Weber, 2014). Ziem (2015) and Wengeler and Ziem (2014) provide a detailed insight into argumentative elements of crisis discourses. Their findings explicitly relate to Lakoff and Johnson (1980) in pointing out that in times of a crisis, cross domain mappings occur with the purpose to map a complex problem to a familiar target domain for efficient decision making. Frequent target domains of those metaphors cover natural disasters as observable reference processes alongside socio-technical metaphors for causal-functional aspects of the crisis. Applying those naturalisation approaches, affected objects, temporal phases of the crisis, consequences and potential profiteers become visible. Metaphors of war and fight help to foster mitigation measures, whereas crises framed as diseases - even in the case of political or economic crisis - relate to the original sense of crisis in health as a deciding moment, in which the outcome of the crisis is determined. The personification of a crisis allows for the usage of the immense human capability to assign motivations, goals and intentional actions in a search for counteractions. The use of metaphors is common in economic crises (O’Mara-Shimek et al., 2015) and even a deliberately set rhetoric means in politics (Charteris-Black, 2011).

In the context of spatial metaphors, our study can already take profit from exploratory studies on the COVID-19 pandemic. First hand, one might expect the pandemic to be a *capsular place* not attached to any specific location (Bissell, 2021). In framing the pandemic as a rapid process, war metaphors were very productive, both indicating a connotation of seriousness and urgency (Semino, 2021) and a call for defensive actions and victory conditions (Chapman and Miller, 2020). At least in the beginning of the COVID-19 pandemic, common defensive tactics of *othering* known from diseases like AIDS (Craig, 2020) were applied to attach the disease to racial or regional attributes (Kremer, 2020). But other than spatial metaphors used in the context of diseases like cancer (*spread*), the war on COVID seams to be primarily structured temporally (*waves*, see Craig, 2020). The main fallacy using war metaphors in this context is to frame the pandemic as an inevitable external threat without means of prevention. In response, Semino (2021) proposes to discuss the alternative metaphor of fire fighting that involves phases of different measures, but does not account for asymptomatic transmission either.

On second visit, although, the intense use of terms like hotspot, break-outs or wildfires refers to metaphors of spatial bound natural disasters that run rapidly and on a large scale (Bissell, 2021). Looking deeper into the spatiality of COVID-19 metaphors, Brinks and Ibert (2020) propose to use the TPSN-Framework (Territory - Place - Scale - Network) of Jessop et al. (2008) to account for the spatial granularity of measures taken. Wearing a mask, for example is bound to action networks at certain places (e.g. supermarkets), whereas contagion measures are bound to the territoriality on the legislative regions (Brinks and Ibert, 2020).

## Explorative pre-study

Based on the findings of these previous studies, in our own qualitative pre-study, we were able to observe several recurring spatial elements of argumentation to be productive in the discourse on the COVID-19 pandemic. The findings are also based on the DWDS-corpus mentioned above and are used to solidify our theoretical vantage point for a later detailed empirical analysis of our corpus (see Section “Methodology and study setup for distant reading approach”).

### Toponyms

Toponyms (names of places) are the most basic way to link semantic meaning and space (for geographical studies on toponymy, see: Alderman, 2008; Rose-Redwood and Alderman, 2010; Scharloth, 2022). In fact, toponyms are a pre- or non-metaphoric form of spatial reference, since they simply address particular positions or areas on the earth’s surface by individual names. In this sense, geography constitutes a basic, non-dispendable layer of meaning in making sense of the pandemic. To determine its origin, to define events and developments in the pandemic means to pin them down on a mental map by the use of place names. Combined with a temporal dimension, toponyms allow to construct easy-to-grasp narratives on the pandemic, making its expansion and network-like global spread imaginable. In doing so, the geographic narrative links several scale levels from local, regional to global – corresponding with different stages of the expansion of COVID. For example: “Many citizens of *Ischgl* were infected” (DWDS/Handelsblatt, 2020) or: “*Ischgl* with its Aprés-Ski-Bars is considered to be a hot spot for the spread of the Corona-virus in Austria and in parts of Europe” (DWDS/Handelsblatt, 2020). Beyond naming places of transmissions, place names are used to determine arenas of discursive and political negotiation of the pandemic and its mitigation measures, as in: “Berlin declares a curfew for shops” (DWDS/Der SPIEGEL, 2020).

In media’s comments on the viruses spreading across the world a convergence of spatial and temporal reference can be observed. Toponyms become synonymous for a particular event which happened at that place. In order to prevent future incidents particular toponyms function as a warning sign: “Northern Italy is the second Wuhan” (DWDS/Handelsblatt, 2020) or, as the secretary of state for health of Germany elaborated in more detail: “we have to be very careful that the “Ballermann” [a party location on Mallorca] does not become a second Ischgl” [a skiing resort in Austria which was an early hotspot of the pandemic in Europe](DWDS/Handelsblatt, 2020).

In sum, toponyms are a crucial element in addressing what the virus actually is and the logic of events in the pandemic in terms of its distribution and dynamic.

### Containerisation and spatialisation

More abstract spatial metaphoric or schematic patterns in the concept of COVID are container schemas, which imagine the virus to be contained by or crossing territorial borders. Spatial containers and their real or imagined boundaries provide the basis for spatial argumentation and reasoning - implicitly linked to concepts of containment as the logical spatial response to the pandemic:

Source 2: “126 cases were recorded in Wuhan, a city in central China, which is considered to be the epicentre of the virus.” (DWDS/Handelsblatt, 2020)

Seemingly purely descriptive in character, the quote shows a container-subcontainer pattern leading from individual cases, to the city of Wuhan to central china. Containerisation provides an immediate understanding of the phenomenon at the cost of generalisation or even stigmatisation of groups of people imagined to inhabit a (sub)container. Containerisation as an ambiguous form of simplification literally allows to “sort things out”. Furthermore, the sentence includes with the seismic metaphor of “epicentre” a naturalisation metaphor to let the relationships between different spatial containers become more tangible (on naturalisation, see below).

Further patterns of spatialisation include concepts which highlight important places in the progress of the pandemic. For example, COVID can be located in particular regions of crisis [German: “Krisenregion”], spreading at “hot spots” or originating from “epicentres” which lead to the implicit construction of *centre-periphery-patterns* in representing the pandemic. With its selecting effects – which places are especially concerned with the crisis and which are not – containerisation and spatialisation determine degrees of social identification and consternation.

### Naturalisation and metaphors of natural disaster

At first sight, imagining COVID as a natural phenomenon appears not as a remarkable metaphoric transfer since it actually is a biological entity. But many of the concepts of nature used to refer to the COVID pandemic strongly differ from the original concept of “virus” and “pandemic”. For example, very common in media discourse is the representation of the COVID-19 pandemic by the use of phrases such as “the perfect storm”, “spreading like wildfire”, a “tsunami”, and one or several “wave(s)”:

Source 3: “We consider the Covid-19-tragedy as a tsunami which is moving from east to west along with economic lock-downs.” (Board Chairman Johann Rupert, DWDS/Handelsblatt, 2020)

To assume an entity to be “natural” means to imply it to be “non-human” in the basic sense of “non-cultural”, and thus, external to humanity. Moreover, with regard to catastrophic events the “natural” is presumed to be an opaque power to humans giving the impression of a random, physically powerful appearance, uncontrollable by man, determined only by its inner natural laws. Thus, public attention may turn away from particular individual’s and institution’s responsibility.

While the effect of generating attention towards the pandemic may be achieved by envoking such dramatic imaginations of a tangible catastrophic physical power upon humankind the ontological dissimilarities between, e.g., a “perfect storm” and a virus may also mislead sustainable healthcare management both in present and future. As Brandt and Botelho put it:

“This language creates a public health discourse that seems reactive rather than proactive, reductive rather than holistic, disempowering rather than empowering. Though its inherent drama may be appealing, the term “perfect storm” invokes notions of randomness and volatility that may actually undermine our ability to address the Covid-19 pandemic and future disease outbreaks.” (Brandt and Botelho, 2020, pp. 1493f).

Especially the metaphor of “the perfect storm” suggests a dramatic, yet short event, which takes place externally and independently from human everyday action. Both attributes prevent people from applying adequate sustainable health care strategies, e.g. to pay attention to simple individual routines over a long time period.

### Personification

To imagine a non-human entity as a person is a metaphor which is, as expected, very common in addressing the coronavirus (see Section “Speech act, pragmatics, and argumentation”, Example 1, above). Turning the virus into a human being implies assumptions on its intelligence (especially its ability to adapt to different surrounding conditions), autonomy (we do not know what the virus will do next), its strengths and weaknesses (we can detract its livelihood), its intentionality and even its rationality or emotional state (the virus rages). It is assumed to act according to its own goals and it responds to our actions with its own. As a Bavarian politician puts it: “If we know how the virus is acting, we have to do everything to slow down its further spreading” (Bavarian State Secretary Florian Hermann, Merkur, 2020; see also the detailed analysis of the argumentation structure above). It can be read as an attempt to promote a deeper understanding of the virus and it suggests an inner complexity of the virus which, yet, has not been fully comprehended by man.

It is easy to see that the virus-as-person metaphor is not a fully adequate representation. But simply criticising this metaphor to be “wrong” because a virus is not a person misses the point. There is no language without metaphor; thus, one has to ask in which regards the metaphor is helpful to communicate particular aspects of the phenomenon and, on the other hand, in which regards it is misleading because it suggests an inadequate representation of the phenomenon for public discourse and political decisions. For example, to paint the dramatic picture of a cruel and reckless enemy of humankind may mobilise people to pay attention to the virus in their everyday lives while, at the same time, it prevents people from gaining a deeper biological understanding of the functioning of viruses in a “non-human” way.

The-virus-is-a-person metaphor allows people to attach several attributes to it – as some kind of mind map – and therefore enabling them to make sense of a sensually nearly non-approachable entity. Its various facets can be imagined, but may be also misrepresented as flaws and moods of a human individual (when projected towards the virus).

Personification is also a crucial element in the spatial argumentation on proper respondings on the pandemic and the distribution of responsibilities in its aftermath. Especially, territorial entities like regions, states and nations are considered to be persons or actors as well – as in the following proposition: “Now the region is fighting an explosion of case numbers” (DWDS/Handelsblatt, 2020).

The notion of territorial entities becoming actors is also established in public international law which constitutes the context for the following example where American attorneys are announcing a lawsuit against China as the presumed culprit and the origin of the corona pandemic: “we have to prove that China acted negligent or even malicious” (DWDS/Handelsblatt, 2020). In this case it is claimed that China can be held responsible for having failed in containing the virus on its territory and giving false information on its spreading. Thus, China is liable and can be sued under American law.

Different patterns of spatial argumentation can be combined such as toponyms, the personification and the naturalisation metaphor in the following example:

Source 4: “Latin America is the new epicentre of the Corona pandemic. Increasing numbers of infections and many lethal cases: The Corona virus is spreading across Latin America like wildfire, an end to this is not in sight. Not every country has responded well to the pandemic, not all countries are equally affected.” (DWDS/Neue Züricher Zeitung, 2020)

In its semantic content personification metaphors appear remarkably different from, in some regards even oppositional, to strategies of “naturalisation”. While “naturalisation” suggests an event to be external and opaque to humans metaphors of “personification” provide a semantic transfer in the opposite direction – the imagining of an actor somehow similar to humans whose actions can be comprehended and, as in the fight against COVID, maybe even foreseen and prevented. However, naturalisation and personification are frequently combined in the media discourse on COVID-19.

In sum, we identified spatial metaphors on COVID-19 to be very productive as a mental model for containment by defining suitable borders (between “us” and the infected ones) that the virus as a person intends to cross on any occasion crushing waves on the shores of everyday routines. In the case of place names, it became clearly visible that only a portion of their usage points to locations of transmission. Beside naming stages of political decision making, place names are often instantly generalised by the argumentative pattern of *zooming-out* (Richter et al., 2013) to identify “infected” crisis regions (e.g. central China) whose adjacent borders had to be closed or at least closely monitored.

As our findings are purely qualitative and punctual at this stage, digital assistance functions can help to learn about effect sizes as well as changes over time on a larger scale. As pure filtering approaches can only help to narrow down corpora on the occurrence of specific query terms, we wanted to combine this approach with structural linguistic patterns indicating use of metaphor and automated semantic context analyses. Whereas measures for context binding (e.g. Moura de Souza et al., 2022) are not sensitive enough to detect interesting individual language usage patterns, they can deliver insights in recurring patterns term cooccurrences.

## Methodology and study setup for distant reading approach

In the broader and well-established context of natural language processing (Jurafsky and Martin, 2018), mining concept relations from texts is a prominent task (e.g. Haarmann, 2014). Yan et al. (2019) propose a method to extract the relational context of place names as knowledge graphs, which includes background knowledge from external databases. On a linguistically less informed level, building on common cooccurrence analyses (Evert, 2009), association rule mining (Hipp et al., 2000) can help to identify close context bindings between terms from texts. In the context of geohumanities, Kremer (2018) has used a generalised approach to derive reference equality between place names and locomotion patterns before.

A lot of effort has been spent to further adapt linguistic methods for the requirements of social sciences (Scholz, 2018). As grounded theory is a well-known approach in social sciences to deduce theory from text (Glaser and Strauss, 2017) that relies on in situ codes only, techniques of knowledge discovery often try to align themselves as computational grounded theory (Lai and To, 2015; Berente and Seidel, 2014; Nelson, 2017). In this context of inductive or hypothesis-generating approaches, Bubenhofer (2009, 2015) proposes to look for recurring language usage patterns in the environment of specific search terms. In his approach, language usage patterns are defined by collocations, i.e. an exact sequence of a specific number of words. For example, ‘bus from Bielefeld’ is a 3-word collocation. These language usage patterns can include part of speech tags as variables, e.g. by setting the third constituent to a proper noun in general. He shows for the specific context of ‘Kampf’ (war) that these approaches form a solid base for an explorative analysis of metaphor as well as argumentation patterns (Bubenhofer, 2009, p. 431). Other than collocations, cooccurrences generalise form a specific word sequence by considering all terms in a certain word window (e.g. a sentence or a paragraph) to be collocated (Evert, 2009). In the sense of a bag-of-word approach this can be generalised further from the grammatical structure of a sentence to a set or list of lemmas occuring in the sentence (Jurafsky and Martin, 2009). Dammann et al. (2021) propose an adaptation of cooccurrence analyses in the context of geo-humanities.

**Fig. 2:**
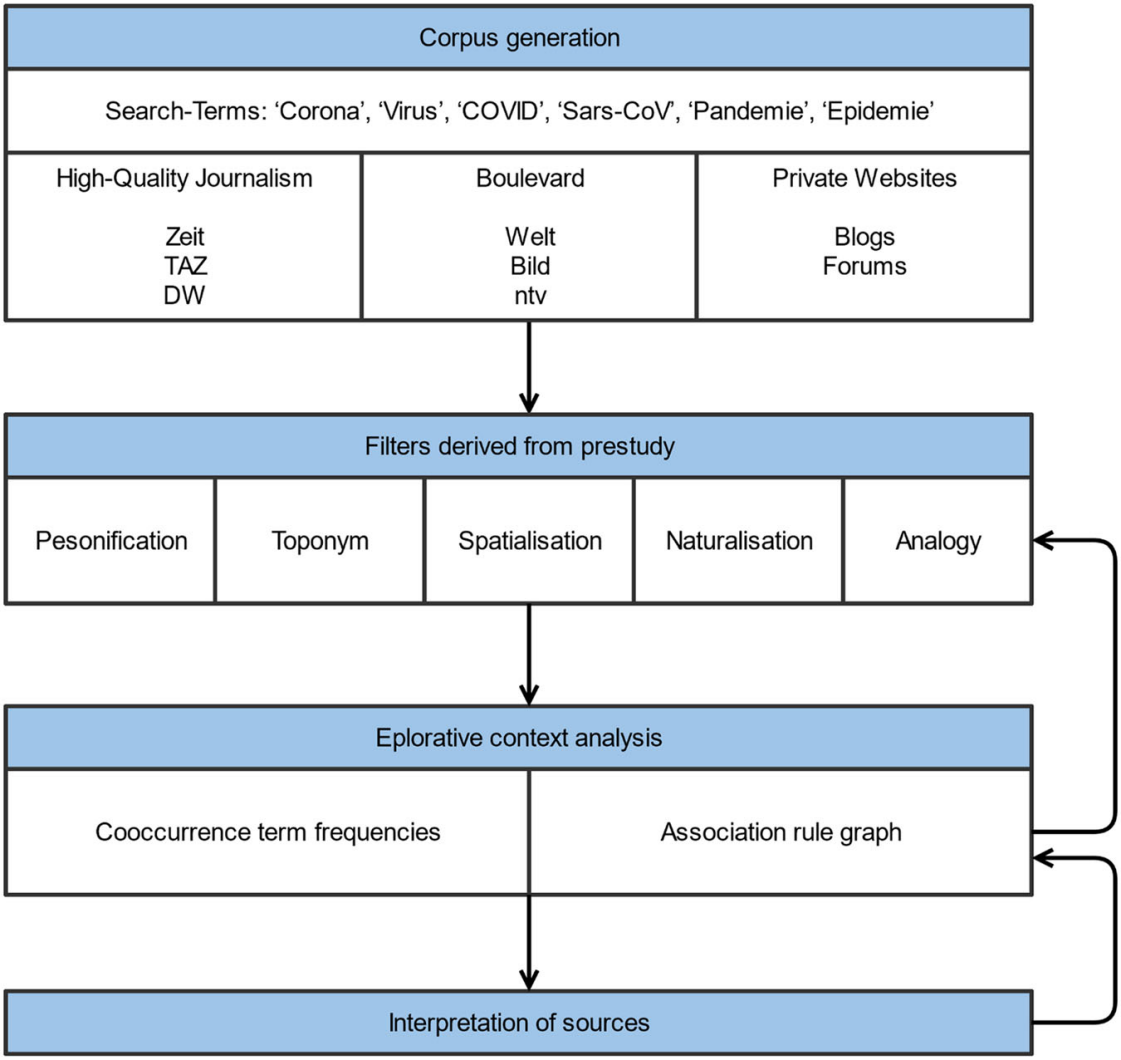
General approach of the method applied conducting this study. The predefined corpus is filter for search terms indicating reference on the COVID-19 pandemic. The resulting collection is mined for the pre-identified language usage patterns indicating use of spatial metaphor. After explorative automated analysis resulting in directed association graphs, interesting sources are interpreted in detail by the introduced scheme derived from Toulmin (1976 [1958]). This figure is covered by the Creative Commons Attribution 4.0 International License. Copyright © the authors of this paper, all rights reserved.

Stefanowitsch and Gries (2007) provide a thorough introduction into (non-digital) corpus-based analysis of metaphor. Traditionally, this is done by identifying source and target domain vocabulary (Stefanowitsch, 2007). In a more generic step, texts can be searched for patterns in the microcontext of metaphors (Hilpert, 2007), e.g. specific lexicalised word sequences or frequently cooccurring terms. In related automatised scenarios on large corpora, methods of unsupervised knowledge discovery from text are often combined with complementary, e.g. qualitative methods (Meng and Shyu, 2012) for validation purposes. As recommended by Stefanowitsch (2007), we therefore use an iterative approach for the exploration of our data (see, Fig. 2):

1. A qualitative exploration informs the search process by identifying suitable search terms and patterns.
2. In a semi-automated distant reading (Moretti, 2013) approach, we compute frequent terms that occur in the semantic context of these search terms as well as structural patterns indicating the use of metaphor.^5^
3. From these frequent co-occurring terms we select interesting term combinations for further filtering.
4. In a concluding step we enrich our initial findings with focussed context graphs on the chosen term combinations for visualisation of micro-contexts and derive additional example statements.

As we wanted to learn more about the variety of use of spatial metaphors in the early stages of the COVID-19 crisis, we used a COVID-19 corpus of the Digital Dictionary of German Language (DWDS^6^) introduced above which has been specifically composed for scientific purposes. To stick as closely as possible to the original debate at that time and to assure availability of the references to websites contained in this corpus, we used a local dump of the corpus retrieved on 2020-08-13^7^. As the COVID-19 corpus of the DWDS allows for live filtering in the web interface as well, this enabled us to follow the mixed-method approach introduced above.

After applying a primary filter selecting only sentences containing ‘Corona’, ‘Virus’, ‘COVID’, ‘Sars-CoV’, ‘Pandemie’, or ‘Epidemie’ or their lowercase counterparts as substrings, we further reduced the corpus to three types of sources to look for differences in subsequent analysis: High-quality journalism, boulevard, and private websites (blogs and forums). At this point our corpus still consisted of 89276 sentences (see, Table 2). Despite the corpus included references to tweets, social media was excluded, as we wanted to look for argumentation patterns instead of memes or deliberately set neologisms.

**Table 2:** Subcorpora selected for comparative analysis.

	High-quality journalism	Boulevard	Private websites
Selected subcorpora	• Zeit (4828) • TAZ (28902) • DW (19331)	• Bild (547) • Welt (7741) • NTV (25158)	• Blogs (2466) • Forums (303)
#sentences	53061	33446	2769

For comparison of the amount and quality of metaphors used, we compiled subcorpora for high-quality journalism, boulevard and private websites.

In congruence with our results from the qualitative exploration step, we then derived indicators for the four identified narrative types of spatial metaphor: personification, toponyms, naturalisation, and spatialisation. In addition, we introduced a fifth pattern representing analogy indicated by comparison particles (see Table 3).

**Table 3:** Search patterns derived from pre-study.

Search pattern	Type of analysis	Condition
Personification	Structural collocation	Part of speech tag sequence equals <COVID>^a^+<VVFIN>^b^+ or <VVFIN>+<COVID>+
Toponym	Named entity recognition	Named entity tag equals ‘LOC’
Spatialisation	Search term list	[‘Grenze’, ‘Region’^c^]
Naturalisation	Search term list	[‘Epizentrum’, ‘Hotspot’, ‘Sturm’, ‘Tsunami’^d^, ‘Welle’]
Analogy	Structural collocation	Part of speech tag sequence equals <COVID>+<$,>?<KOKOM>^e^?<NE|NN>+ or <NE|NN>+<$,>?<KOKOM>?<COVID>+

According to the findings of the pre-study, we operationalised each of the language use patterns by either a list of search terms or a syntactic criterion building on part-of-speech tags.

^a^Occurrences of the terms ‘Corona’, ‘Virus’, ‘COVID’, ‘Sars-CoV’, ‘Pandemie’, or ‘Epidemie’ were marked by a new artificial part of speech tag ‘<COVID>’.

^b^Finite verb form, cf. TIGER Project (2003).

^c^Due to a lack of additional information, ‘Region’ was dropped on further exploration.

^d^Due to a lack of additional information, ‘Tsunami’ was dropped on further exploration.

^e^Comparison particle, cf. TIGER Project (2003).

**Fig. 3:**
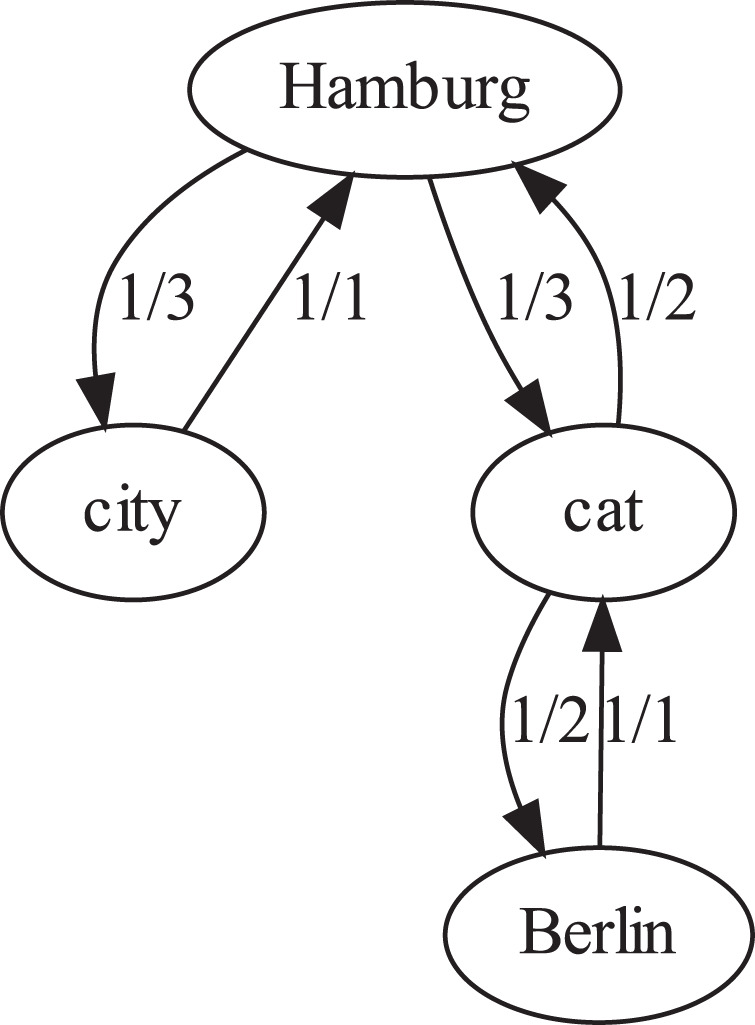
Example graph yielded by association rule mining. An example graph visualises the result of the rules extracted by association rule mining on the introduced text fragment. Nodes represent (proper) nouns, edges are annotated with the frequency of the originating node in all sentences containing the target node. This figure is covered by the Creative Commons Attribution 4.0 International License. Copyright © the authors of this paper, all rights reserved.

These search patterns were applied to the selected subcorpora. To allow for further qualitative analysis, we first reduced the cooccurrence context of our patterns and search terms to (proper) nouns, and then applied an explorative graph visualisation by association rule mining (Hipp et al., 2000) on top of it. Figure 3 illustrates the process using a simplified fictional example:

‘We live in Hamburg. We find Hamburg to be a nice city. Our cat is with us in Hamburg. The cat ran away to Berlin.’

‘Hamburg’ occurs in three sentences, ‘cat’ in two, and ‘Berlin’ and ‘city’ in one. Association rules are then computed based on cooccurrences: because ‘cat’ occurs alongside one of the three occurrences of ‘Hamburg’, the term ‘Hamburg’ points with an estimated confidence of 0.33 to ‘cat’. However, as ‘Hamburg’ occurs in only one of the two occurrences of ‘cat’, the term ‘cat’ is bound to the context of ‘Hamburg’ with an estimated confidence of 0.5. This definition of context binding, when deployed on larger text corpora, can thereby provide useful automated identification of contextual linkages and point to argumentative elements in bodies of text (Moura de Souza et al., 2022).

As we looked for the variability of environments more than confident recurring patterns, we configured our tool to include terms with a frequency of 3 and more and association rules with a confidence of 0.1 or above. As a result, we were able to use the filter patterns in combination with exploration of their semantic context as a combined filter-refine approach (Wood, 2008) to quickly explore large amounts of text for occurrences of the pre-identified language use patterns.

## Results from the distant reading approach

In this section, the obtained results for each of our pre-identified fields of interest are presented. More specifically, we show (1) results of frequency analyses of the semantic contexts obtained by the filter methods above, (2) semantic context graph visualisations of ambigue samples further limited by adding frequent or interesting search terms and (3) interpretation of identified relevant single statements discovered thereby. For comparison within the preselected corpus, we use our pre-compiled corpora to look for differences between high-quality journalism, boulevard, and private websites. The underlying assumption to be tested was that less critical or reflective reports on the pandemic tend do use more figurative language including metaphors and that private websites tend to contain more unique metaphors than the well-coordinated language of press media. We were able to observe this at least for specific contexts while our selective approach does not allow for generalisation.

### Personification

**Fig. 4:**
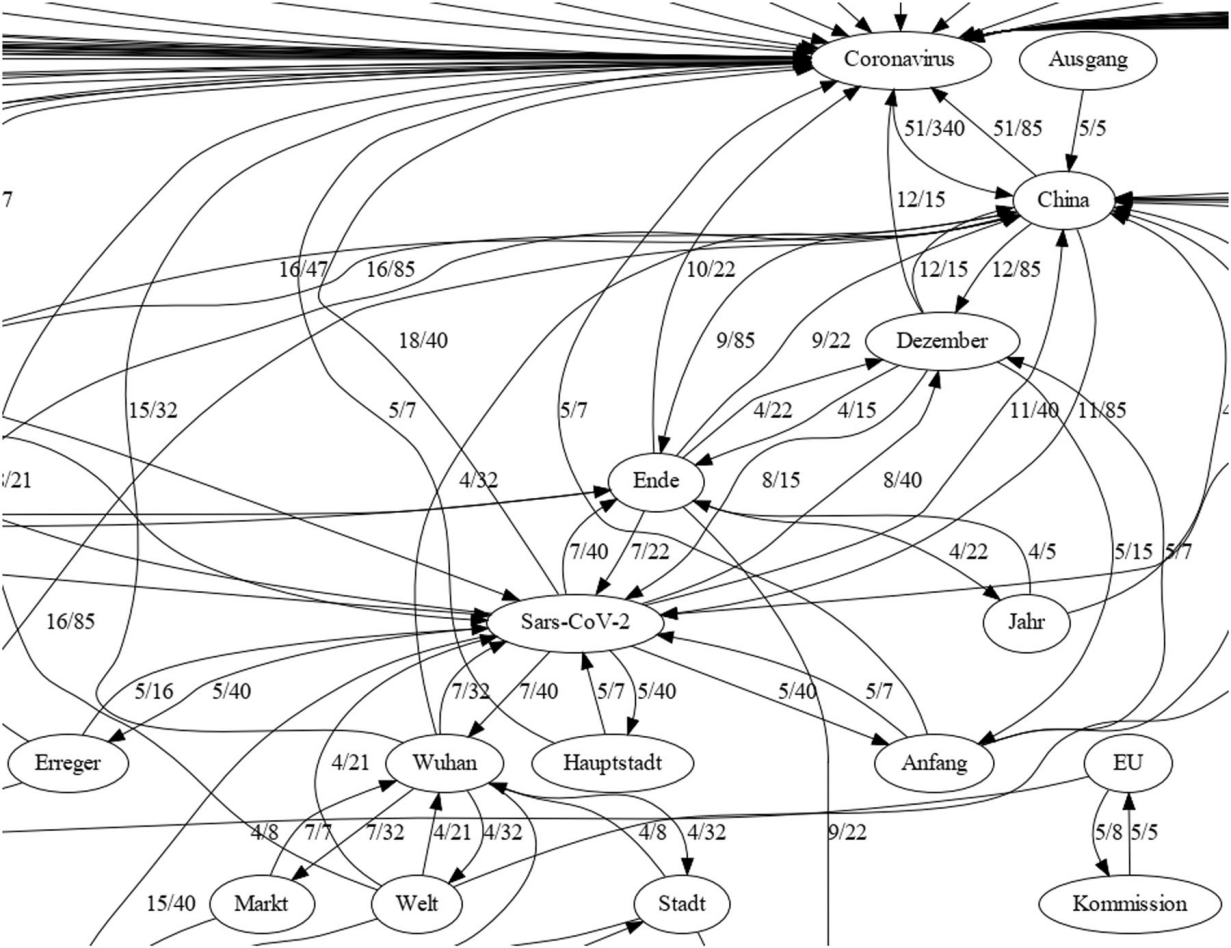
Detailed semantic context analysis. Clipped part of a context binding graph in the semantic context of the additional search term ‘breiten’. As introduced above, values assigned to directed edges indicate the strength of contextual linkages, e.g. 5 of the 5 occurrences of ‘Kommission’ share the context with ‘EU’ which binds the first term completely to the scope of the second one on all occurrences in the pre-set context. This figure is covered by the Creative Commons Attribution 4.0 International License. Copyright © the authors of this paper, all rights reserved.

Indicating COVID-19 to be acting as a person by extracting verbs in direct collocation with one of the terms pointing to the COVID-19 pandemic obtained the following result. Besides the interesting notion of change (‘verändern’) and challenge for (re-)acting (‘handeln’) on private websites the single spatial metaphor clearly used across all texts was the spread of COVID-19 (‘(aus)breiten’). In a context binding graph of the boulevard press filtered on ‘breiten’, we were able to visualise the condensed context bindings of the core narrative about the origin of COVID-19 (see, Fig. 4). An example sentence shows how the components are arranged on recurring occasions:

Source 5: “The world’s first cases of infection with the new coronavirus were reported from the industrial metropolis at the end of December. The capital of the province Hubei was the first Chinese city to be completely closed off due to the spread of the virus on January 23rd, which was later followed by almost the entire province.” (DWDS/Welt, 2020)

### Containerisation and localisation

#### Toponyms

Toponym analysis in our sample reveals that the public on COVID-19 was initially almost exclusively bound to the national level (see, Table 4).

**Table 4:** Count of most frequent toponyms.

	High-quality journalism	Boulevard	Private Websites
Frequent spatial terms	• Land (997)	• Land (708)	• Land (24) • Welt (19)
Frequent toponyms	• Deutschland (2639) • China (1598) • Italien (941) • USA (835) • Europa (734) • Berlin (582) • Wuhan (544)	• Deutschland (1672) • China (1458) • USA (817) • Italien (712) • Wuhan (592) • Berlin (502) • Europa (495)	• Deutschland (66) • China (43) • Italien (30) • Europa (22) • USA (17) • Wuhan (16)

For each of the comparative subcorpora we list the most frequent occurrences of spatial terms and toponyms in the selected sentences.

The following example shows that the national level is the primary level of affectedness as well as the primary level of measures taken:

Source 6: “Meanwhile Spain counts even more coronavirus deaths than China: according to the Ministry of Health in Madrid on Wednesday, 3434 people have died from the lung desease covid-19. After Italy – meanwhile counting 6820 coronavirus deaths – Spain is the most affected country by the coronavirus pandemic in Europe. Despite the strict curfew enforced eleven days ago, the number of infected people rose to 47610.” (DWDS/Welt, 2020)

When looking for reasons for the pandemic the first reflex of national action was combined with the reflex to represent certain places as spatially bound root causes of the pandemic. The spatially defined origin of the virus is presented as its actual cause:

Source 7: “Allerberger stated that currently 600 cases of corona infection in Austria can be traced back to Ischgl and the surrounding communities.” (DWDS/Welt, 2020)

Detail analysis 3: Spatial reference as presumed causation. 

The association of the national level and immediate action is expectable, because quick and conform legislative action is bound to this level. Besides another use of toponyms as metaphors themselves (“second Ischgl”), we find it remarkable that in the early stages of COVID-19, place names provided a frequently used means to reason that almost all transmissions of COVID-19 could be explained by events at certain places.

#### Border (Grenze)

Besides toponyms and pandemic-related terms, among the top cooccurring terms for *border* is *measures* (‘Maßnahmen’) which refers to borders as well as *hotspots* as concepts deemed suitable to govern the crisis. In addition, but in a lexicalised sense in German, ‘Grenze’ is frequent as part of ‘Obergrenze’ referring to the *threshold* of weekly COVID-19 infections which were used to define when more strict rules had to be put in effect.

**Fig. 5:**
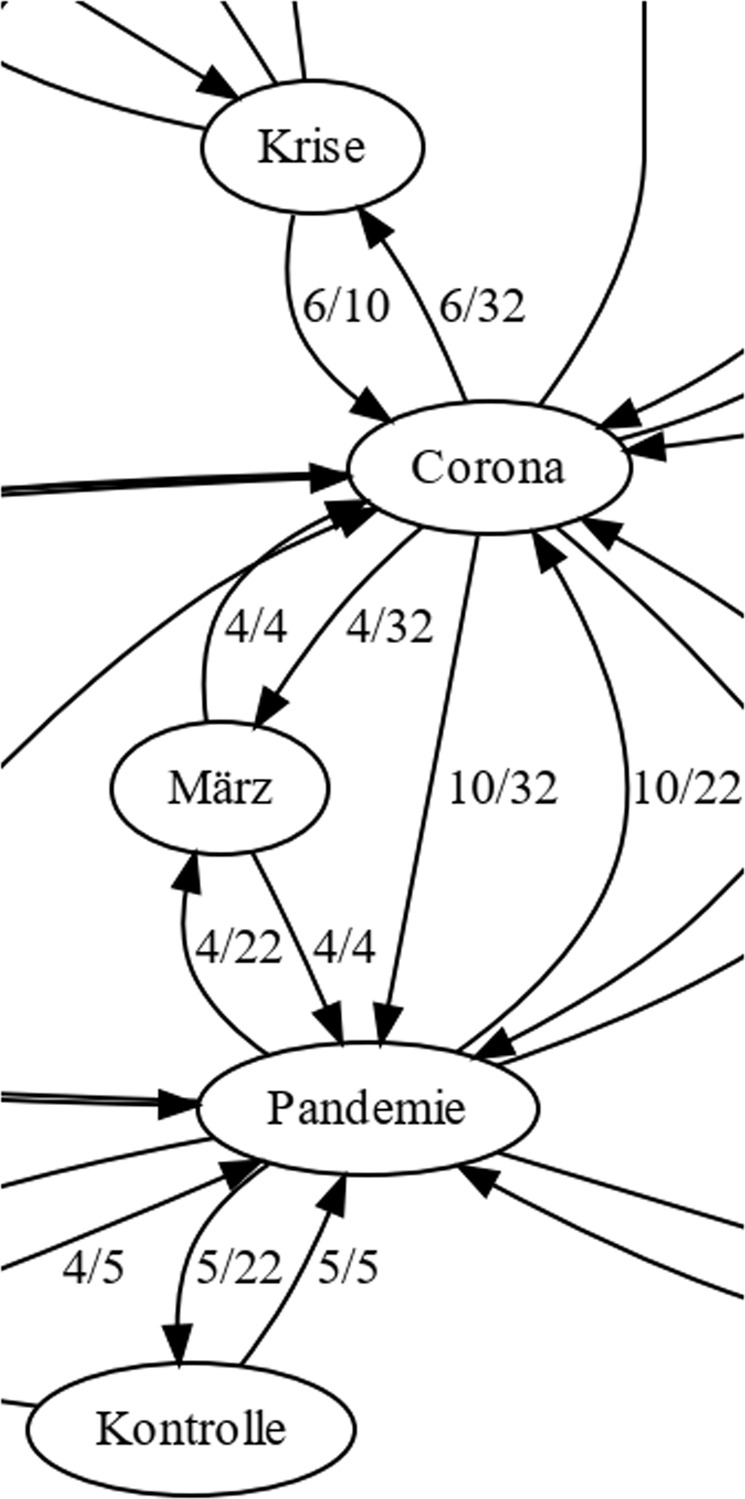
Detailed semantic context analysis. Recurring pattern of term binding in the context of ‘Grenze’ (border) and ‘Maßnahmen’ (measures) in boulevard media (clipped graph area). Strong mutual dependencies are observed between occurrences of ‘Krise’ (crisis) and ‘Corona’. ‘Kontrolle’ (control) is an important context marker of ‘Pandemie’ (pandemic). This figure is covered by the Creative Commons Attribution 4.0 International License. Copyright © the authors of this paper, all rights reserved.

From a detailed semantic context analysis of the filter COVID-synonym, ‘Grenze’ (*border/threshold*), and ‘Maßnahme’ (*measures*) we were able to detect a recurring sequence of context bindings highlighting the demand for (spatial) control in that situation (Fig. 5):

In an explicit metaphoric use, borders refer to the limited capacity of public health services:

Source 8: “Experts are concerned that there could be a strong increase in the number of cases, which will push the public health departments to their limits in tracing chains of infection.” (DWDS/Bild, 2020)

### Metaphors of natural disaster

#### Hotspot

The semantic context of ‘Hotspot’ (upper case as a German term) proved to be a very productive metaphor to describe the site of action and involved many toponyms for that purpose. In contrast to the findings on toponyms alone, hotspots were associated more frequently on the scale level of cities and municipalities (Heinsberg, Mitterteich, New York, Ischgl) than regions or nations. This is a congruent finding with the determination of root causes mentioned above. The tendency to pin the pandemic to certain places was (proportionally) mentionably larger in the boulevard press than in high-quality journalism (Table 5).

**Table 5:** Count of most frequent toponyms in the context of ‘Hotspot’.

	High-quality journalism	Boulevard	Private websites
Frequent spatial terms in the context of ‘Hotspot’	• Stadt (6) • Gemeinde (4)	• Stadt(gebiet) (13) • Land (5) • Region (3) • Ausland (3)	• Skiregion (1)
Frequent toponyms in the context of ‘Hotspot’	• USA (14) • Heinsberg (9) • Spanien (5)	• Spanien (9) • USA (5) • Mitterteich (4) • Deutschland (4) • New York (4) • Ischgl (3)	• Deutschland (1)

In comparison to Table 4 we list the most frequent occurrences of spatial terms and toponyms in sentences containing the term ‘Hotspot’.

Citations of policy makers indicate that in addition to the general regulations on the national level, hotspots provided the main unit of thought to interrupt transmissions on a spatially more fine-grained level. Interestingly, the solution was still thought to be a spatial one.

Source 9: “Bavarias Minister of the Interior Joachim Herrmann (CSU) has informed himself this evening about the situation in Mitterteich. “After the Coronavirus was found remarkably often in the city of Mitterteich, we have to assume it is a hotspot”, Hermann announced before his visit to the city. To limit the spread of the virus and stop the chain of infection, a curfew is an obvious measure.” (DWDS/Welt, 2020)

Similar to the findings on ‘Hotspot’, the search term ‘Epizentrum’ (*epicentre*) shows similar cooccurring terms with a slightly higher share of the toponyms ‘Wuhan’ and ‘Hubei’ situating the origin of the pandemic in Chinese cities - especially in boulevard press. Only in high-quality journalism, Italy and Europe are framed on a similar level as epicentres. Interestingly, also New York is often framed as an epicentre of the pandemic at that time.

#### Wave (Welle)

In the case of ‘Welle’ (*wave*), the subcorpus of ‘Deutsche Welle’ was excluded from analysis as the term is part of the name of the news agency. The metaphor of the wave proved to be productive not only as overarching theme of the naturalisation of the pandemic, but also in general, e.g. in the motif of the wave of solidarity:

Source 10: “The context is that Johnson received a wave of solidarity during his own corona illness, while being under strong criticism for his initial lurch.” (DWDS/Bild, 2020)

#### Storm (Sturm)

Interestingly, beside the strong metaphor of the *perfect storm* in the English speaking world, looking for ‘Sturm’ revealed to not be very productive in our corpus (see, Table 6).

**Table 6:** Count of frequent context terms in the context of ‘Sturm’.

	High-quality journalism	Boulevard	Private websites
Frequent context terms of ‘Sturm’	• Noah (2) • Arche (2) • Wunder (2)	• Meerjung- • frauen (2) • Figur (2)	• Ruhe (1)

We list the most frequent terms co-occurring in the context of ‘Sturm’ on the sentence level.

A congruent political metaphor deliberately put against the storm is ‘Arche Noah’:

Source 11: “The construction site of the new hospital is on the same place, where 17 years ago the state media praised at the time as a “medical miracle” or “Noah’s Ark against the storm of the stars-epidemic”. In six days and seven nights up to 7000 construction workers built a huge quarantine-hospital, in which up to one-seventh of all sars-patients were treated over a two-month period.” (DWDS/TAZ, 2020)

#### Metaphor by analogy

Looking for nouns behind comparison particles in the context of COVID-19 revealed repeating usage of several metaphors (see, Table 7).

**Table 7:** Metaphors co-occurring with COVID-19 synonyms (selection).

	High-quality journalism	Boulevard	Private Websites
Nouns which follow comparison particles in collocation with COVID-19	• Heraus-forderung (4) • Brandbe-schleuniger (3) • Bedrohung (2) • Lauffeuer (1)	• Brandbe-schleuniger (4) • Damokles-Schwert (2) • Angreifer (2)	• Corona-Bond-Muezzins (1) • Herausforderung (1)

We list selected metaphors co-occurring in the context of COVID-19 synonyms on the sentence level.

Consistent to ‘Brandbeschleuniger’ (*fire accelerator*) as a productive metaphor in the beginning of the pandemic to mark the adaption pressure on society especially regarding already disadvantaged persons, the metaphor ‘Lauffeuer’ (*spreading wild fire*) is used to indicate that transmissions might be out of control from this point:

Source 12: “’People will die on the streets’, they said. It was feared that the covid virus could ‘spread like a wildfire in the train stations district and beyond’, if there were no immediate actions taken. Not only in Frankfurt’s train stations district, but also nationwide, people that use drugs are especially at risk at suffering a life-threatening course of disease form a covid infection.” (DWDS/TAZ, 2020)

## Discussion

In sum, space has been used as a powerful target domain for reasoning and decision making in early stages of the COVID-19 pandemic. The national container provided the first instance for defining political measures. Given the main metaphor of a virus agent intending to spread, one prominent means was to close borders in order to contain transmissions in certain regions. When this technique of containment proved to be not as effective as planned, e.g. under the condition of asymptomatic transmission, adaptations of the approaches still used the spatial domain to explain and govern the crisis. ‘Hotspots’ were identified not only for the purpose of containment, but also as root causes for all registered infections in other areas (e.g. Ischgl), when transmissions already took place on a dispersed and global scale. In a last instance, Wuhan, the capital of the Chinese province Hubei was recurrently told to constitute the (distant) epicentre of the pandemic, sending shockwaves around the globe. Interestingly, in some cases like New York, hotspots became so prominent that they were framed as epicentres themselves. From single examples taken from media discourse, we were able to show how switching spatial scales by zooming out for generalisation purposes provided means to quickly deduce if contact with other peoples would form a risk.

Even more interestingly, all processes from the domain of natural disasters used as metaphors (earthquakes, waves and spreading wildfires) develop usually very quickly, whereas pandemics in general can be measured in years. It will be subject to further research, if the pandemic was really believed to happen in a high intensity, and quickly, or if the acceleration of the target domain is just a useful cognitive technique for quick imagination and consecutive reasoning.

Conceptually, we proposed the vantage point of argumentation theory to bridge the gap between qualitative discourse analysis and distant reading approaches based on semi-automatic processing of text - informed by cognitive linguistics. Repeating lines of arguments form the core elements of discourses in everyday language that reiterate established discursive settings, but also try to bind rapid developments in times of a crisis - in offering explanations as well as deriving quick countermeasures. As long as these narratives are intact, measures taken are believed to prove that crises can still be prevented from deteriorating into a catastrophe. We believe crises like the COVID-19 pandemic to reveal more about the internal structure of discourses, when they are not enacted in a self-evident fashion, but exactly when their legitimation is at stake.

Methodologically, our semi-automated filter-refinement approach proved to be very useful in combining classical distant reading approaches from text science to quickly identify semantic contexts in which pre-identified patterns of spatial metaphor occur. By slightly generalising language usage patterns to syntactic patterns and using association rule mining as cooccurrence association measure, we were able to conduct a powerful iterative approach to screen and subsequently examine language usage of spatial metaphor in a larger corpus. Although we did not conduct a systematic evaluation of our toolset in terms of information-retrieval measures like recall and precision, we can report that we were able to collect relevant text passages with a similar effort like simply reviewing hitlists from known information retrieval portals. Instead of combining close and distant reading approaches to a single sequence, we call for iterative, interconnected workflows to explore and examine micro-hypotheses in high velocity. Of course, our toolset can be extended any time by additional patterns or search terms in subsequent research.

## Conclusion

In the early stages of COVID-19, an intense use of metaphor to explain and govern the crisis had to be expected. Besides its well-known impact on (geo-)politics, a thorough analysis of the use of spatial metaphors was still missing. In our approach, we rely on the foundational work of Lakoff and Johnson (1980) on image schemata, and prior work on spatial metaphors as part of argumentation patterns from cultural geography (Schlottmann, 2008). In applying Toulmin’s (1976 [1958]) framework on examples taken from the DWDS corpus on COVID-19, we show how space is often conceptualised as concluding justification for political action. In doing so, we propose to bridge discourse analysis and cognitive linguistics with the vantage point of argumentation theory.

In order to combine our qualitative and exemplifying pre-study with distant reading approaches, in a first step, we deduced from related work which metaphor patterns had to be expected in the framing of crisis in general. In a second step, we combined essential collocation and cooccurrence measures from cognitive linguistics to an iterative filter-refinement approach to look not only at recurring patterns, but also especially at the variability of individual efforts to frame the COVID-19 pandemic as manageable crisis.

Our results show that beside the well-known timeframe of pandemics of several years, metaphors from the domain of natural disasters frame the pandemic as intense, but temporally limited. Guiding narratives report the Chinese city of Wuhan as the epicentre of the pandemic, sending shockwaves to hotspots all over the world. In an understandable effort to govern the crisis, containment measures were applied on a national level first, including closing the borders. Even if it became clear that transmissions were not controllable by those measures, politicians continued to explain that current infections would be nearly all bound to ‘super-spreading events’ at certain hotspots at municipality level.

As the manifold examples of our study show, spatial metaphors and their potential to transform the understanding of an entity can either lead to effective communication in situations of urgency and crisis or there can be potential for fallacies due to misleading semantic implications – such as the prevention of sustainable longterm strategies caused by a misrepresentation of crisis in terms of catastrophic, yet, short term natural events. The analysis of spatial argumentation and metaphor, combined with the presented filter-refinement approach, can contribute to better understanding in which cases which effects can be expected and why.

## Supplementary information

Appendix

## Data Availability

All data used for the qualitative pre-study has public availability at https://www.dwds.de/d/korpora/corona. All data used for automated processing and subsequent qualitative analysis was downloaded from the related repository at https://github.com/adbar/coronakorpus on Aug 13 2020.

## Footnotes

1 Hampe (2005, p. 1) defines “image schema” as “…directly meaningful (“experiential”/“embodied”), preconceptual structures, which arise from, or are grounded in, human recurrent bodily movements through space, perceptual interactions, and ways of manipulating objects.” They possess a metaphoric potential since they allow to structure language according to bodily experience and, thus, link direct experience and abstract concepts.

2 DWDS is the publically funded Digital Dictionary of German Language, hosting corpora and information retrieval tools for analysing contemporary German (Geyken et al., 2017). Explorative analysis made use of any data from 2020 as offered in the web interface of DWDS.

3 We decided to present all citations in English translation for an international audience. Original German sources can be looked up in the appendix.

4 In fact, Bielefeld is situated in the German federal state of Nordrhein-Westfalen.

5 Technically, we rely on Qi et al. (2020) and Bird et al. (2009), used in a Python framework

6 Geyken et al. (2017)

7 According to the version of that day retrieved from https://github.com/adbar/coronakorpus.
