# Correction: Reasoning COVID-19: the use of spatial metaphor in times of a crisis

**DOI:** 10.1057/s41599-022-01474-0

**Published:** 2022-12-13

**Authors:** Dominik Kremer, Tilo Felgenhauer

**Affiliations:** 1 Friedrich-Alexander Universität Erlangen-Nürnberg, Erlangen, Germany; 2 Pädagogische Hochschule Oberösterreich, Linz, Austria

**Keywords:** Geography, Language and linguistics

Correction to: *Humanities and Social Sciences Communications* 10.1057/s41599-022-01264-8, published online 08 August 2022.

In this article the affiliation details for Tilo Felgenhauer were incorrectly given as ‘Pädagogische Hochschule Oberösterreich, Linz, Germany’ but should have been ‘Pädagogische Hochschule Oberösterreich, Linz, Austria’. The original article has been corrected.

