# Supplementary material for: Reasoning COVID-19: the use of spatial metaphor in times of a crisis
Source: Humanit Soc Sci Commun. 2022 Aug 8;9(1):265. doi: 10.1057/s41599-022-01264-8 (PMC9360696; doi:10.1057/s41599-022-01264-8)
Supplement: Supplementary file 1 — Appendix [file 41599_2022_1264_MOESM1_ESM.docx]

# APPENDIX

# Reasoning COVID-19: The use of spatial metaphor in times of a crisis

Kremer, Dominik (dominik.kremer@fau.de)

Friedrich-Alexander-Universität Erlangen-Nürnberg, Germany

Felgenhauer, Tilo

Pädagogische Hochschule Oberösterreich, Austria

### Original Sources in German

Example 1: Wenn man weiß, wie dieses Virus agiert, muss man alles tun, um die Ausbreitung zu verlangsamen. (Merkur 2020-03-27)

Example 2: Das Abriegeln ganzer Städte und Regionen sei wirksamer, weil damit auch asymptomatische Überträger weniger Kontakte zu anderen Menschen haben. (Tagesspiegel 2020-03-12)

Source 1: Was macht denn der Bus aus Bielefeld hier? Der soll heim, in Niedersachsen ist doch Corona! (Kremer 2020)

Source 2: 126 der Fälle wurden in Wuhan, der Stadt in Zentralchina, die als Epizentrum des Virus gilt, gemeldet. (Handelsblatt, 2020-12-02)

Source 3: Wir betrachten die Covid-19-Tragödie wie einen Tsunami, der sich mit den wirtschaftlichen Lockdowns von Osten nach Westen bewegt. (Handelsblatt, 2020-12-01)

Source 4: Lateinamerika ist das neue Epizentrum der Corona-Pandemie. Steigende Ansteckungsraten und viele Tote: Das Coronavirus breitet sich in Lateinamerika wie ein Lauffeuer aus, ein baldiges Ende ist nicht in Sicht. Nicht alle Länder haben gleich gut auf die Pandemie reagiert, und nicht alle sind gleich stark davon betroffen. (Neue Zürcher Zeitung, 2020-07-04)

Source 5: Aus der Industriemetropole waren Ende Dezember die weltweit ersten Infektionsfälle mit dem neuartigen Coronavirus gemeldet worden. Die Hauptstadt der Provinz Hubei wurde wegen der Ausbreitung des Erregers als erste chinesische Stadt am 23. Januar komplett abgeriegelt, später folgte fast die gesamte Provinz. (Welt, 2020-04-08)

Source 6: Mittlerweile zählt Spanien sogar mehr Coronavirus-Todesopfer als China: Nach Angaben des Gesundheitsministeriums in Madrid vom Mittwoch starben bislang 3434 Menschen an der Lungenkrankheit Covid-19. Nach Italien – inzwischen 6820 Coronavirus-Tote – ist Spanien somit das am stärksten von der Coronavirus-Pandemie betroffene Land in Europa. Trotz der vor elf Tagen verhängten strikten Ausgangssperre stieg die Zahl der Infizierten derweil auf 47.610 an. (Welt, 2020-03-25)

Source 7: Allerberger erklärte, dass sich derzeit rund 600 Corona-Infektionen in Österreich auf Ischgl und die umliegenden Gemeinden zurückführen ließen. (Welt, 2020-04-02)

Example 3: see source 8

Source 8: Experten sind besorgt, dass es zu einem starken Anstieg der Fallzahlen kommen könnte, der die Gesundheitsämter bei der Nachverfolgung von Ansteckungsketten an Grenzen bringt. (Bild, 2020-08-11)

Source 9: Bayerns Innenminister Joachim Herrmann (CSU) hat sich am Abend über die Situation in Mitterteich informiert. «Nachdem das Coronavirus auffällig oft im Stadtgebiet von Mitterteich festgestellt wurde, müssen wir von einem Hotspot ausgehen», teilte Herrmann schon vor seinem Besuch in der Stadt mit. Mit einer Ausgangssperre die Ausbreitung des Virus einzudämmen und die Infektionskette zu unterbrechen, sei eine naheliegende Maßnahme. (Welt, 2020-08-13)

Source 10: Hintergrund ist, dass Johnson nach einer Welle der Solidarität während seiner eigenen Corona-Erkrankung ohnehin stark wegen seines anfänglichen Schlingerkurses in der Kritik steht. (Bild, 2020-05-25)

Source 11: Die Baustelle des neuen Krankenhauses ist genau der Ort, an dem vor 17 Jahren geschah, was die Staatsmedien damals wahlweise als „medizinisches Wunder“ oder „Arche Noah gegen den Sturm der Sars-Epidemie“ gepriesen haben. In sechs Tagen und sieben Nächten zogen bis zu 7.000 Bauarbeiter ein riesiges Quarantäne-Krankenhaus hoch, in dem über eine Zeitspanne von zwei Monaten bis zu ein Siebtel aller Sars-Patienten behandelt wurden. (TAZ, 2020-02-07)

Source 12: „Menschen werden auf der Straße sterben“, heißt es. Man befürchte, dass sich das Coronavirus „wie ein Lauffeuer im Bahnhofsviertel und darüber hinaus verbreiten“ könne, wenn nicht sofort gehandelt werde. Nicht nur im Frankfurter Bahnhofsviertel, auch bundesweit sind drogenkonsumierende Menschen besonders gefährdet, einen lebensbedrohlichen Krankheitsverlauf bei einer Corona-Infektion zu erleiden. (TAZ, 2020-04-09)
